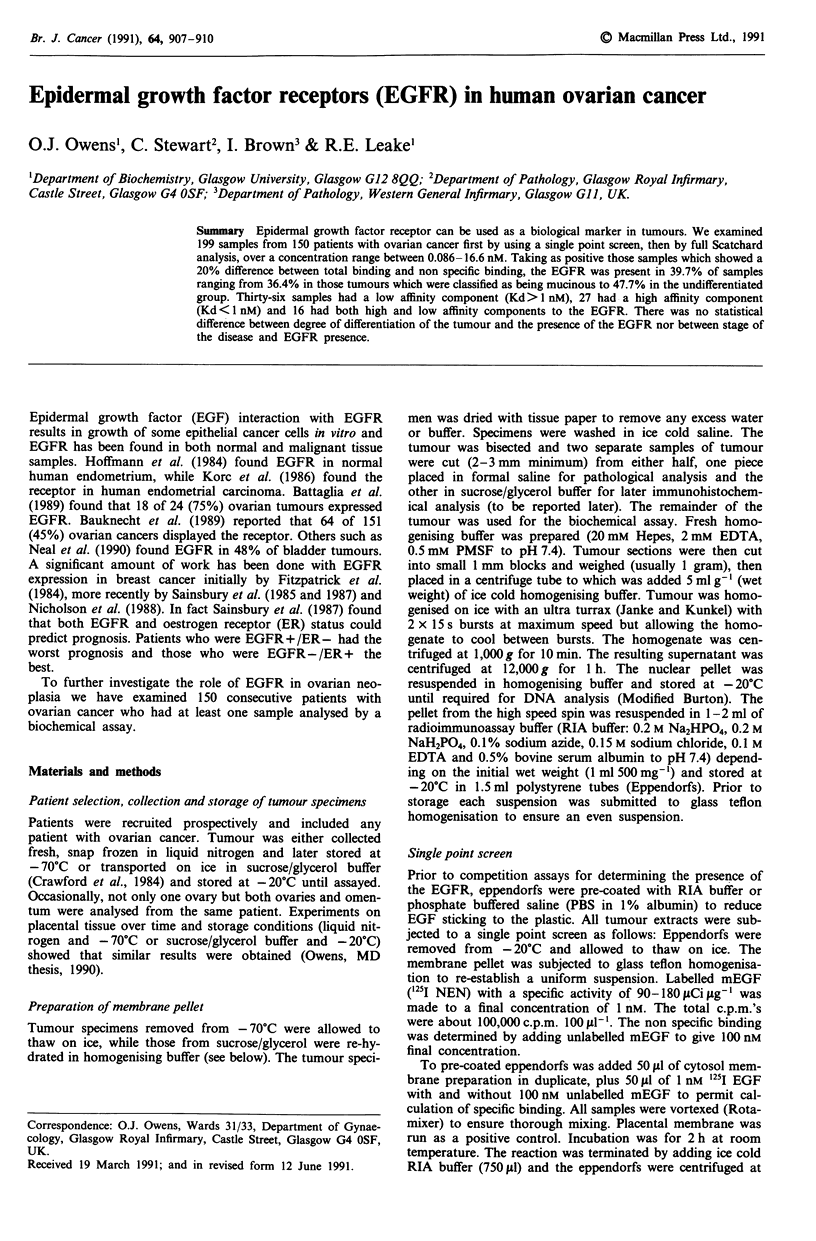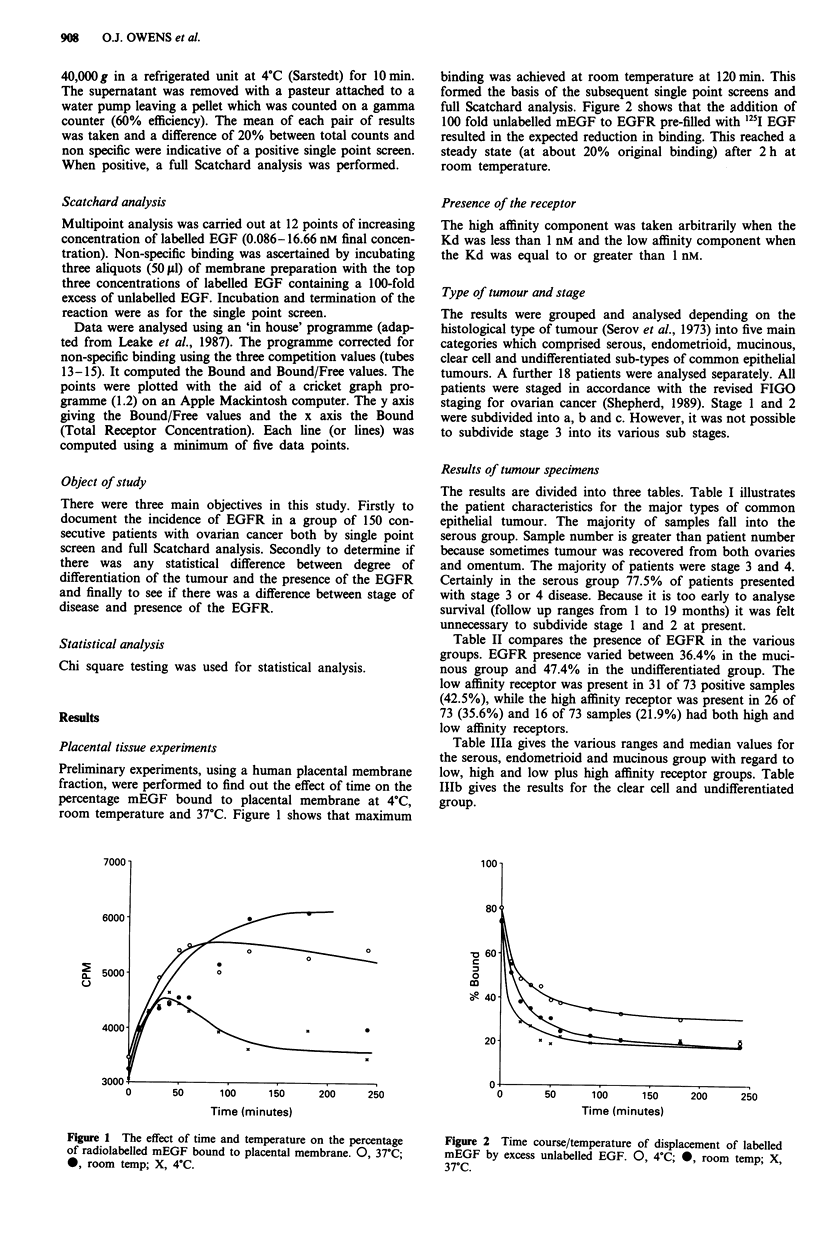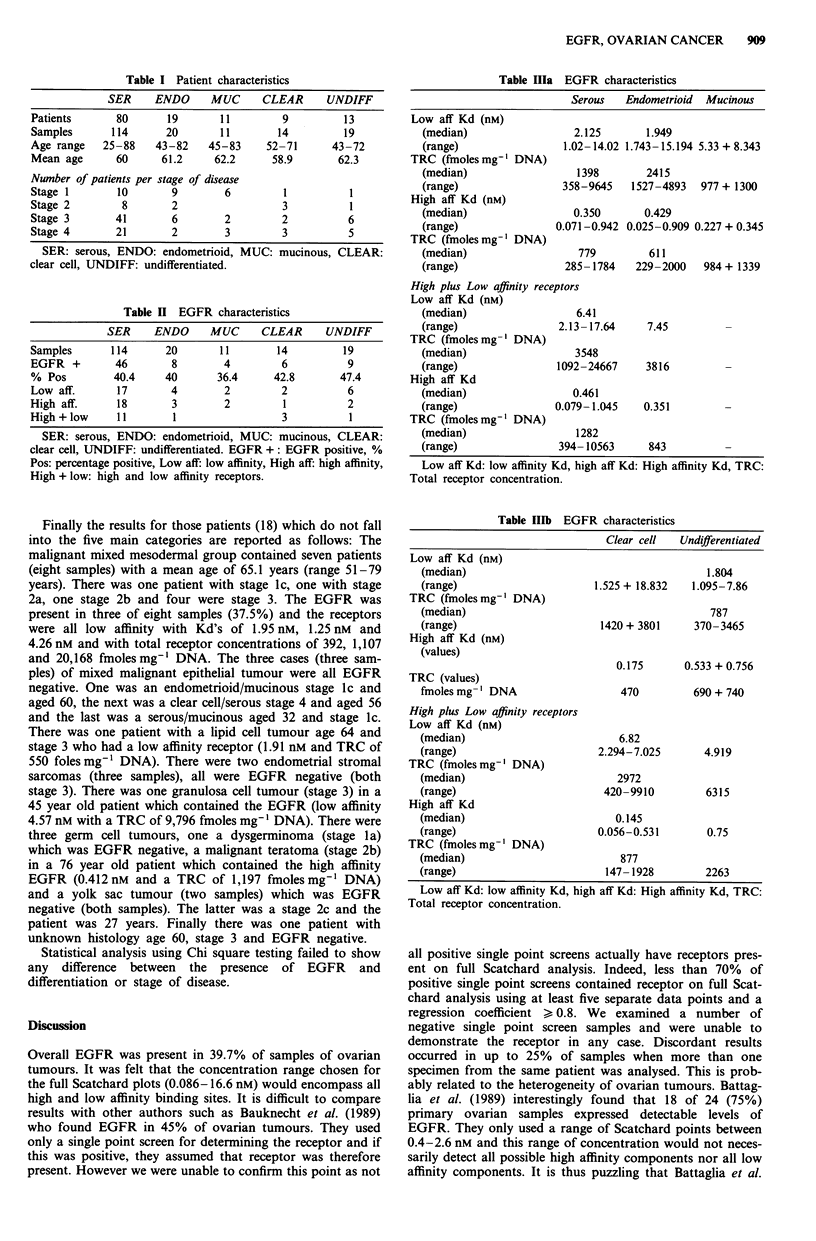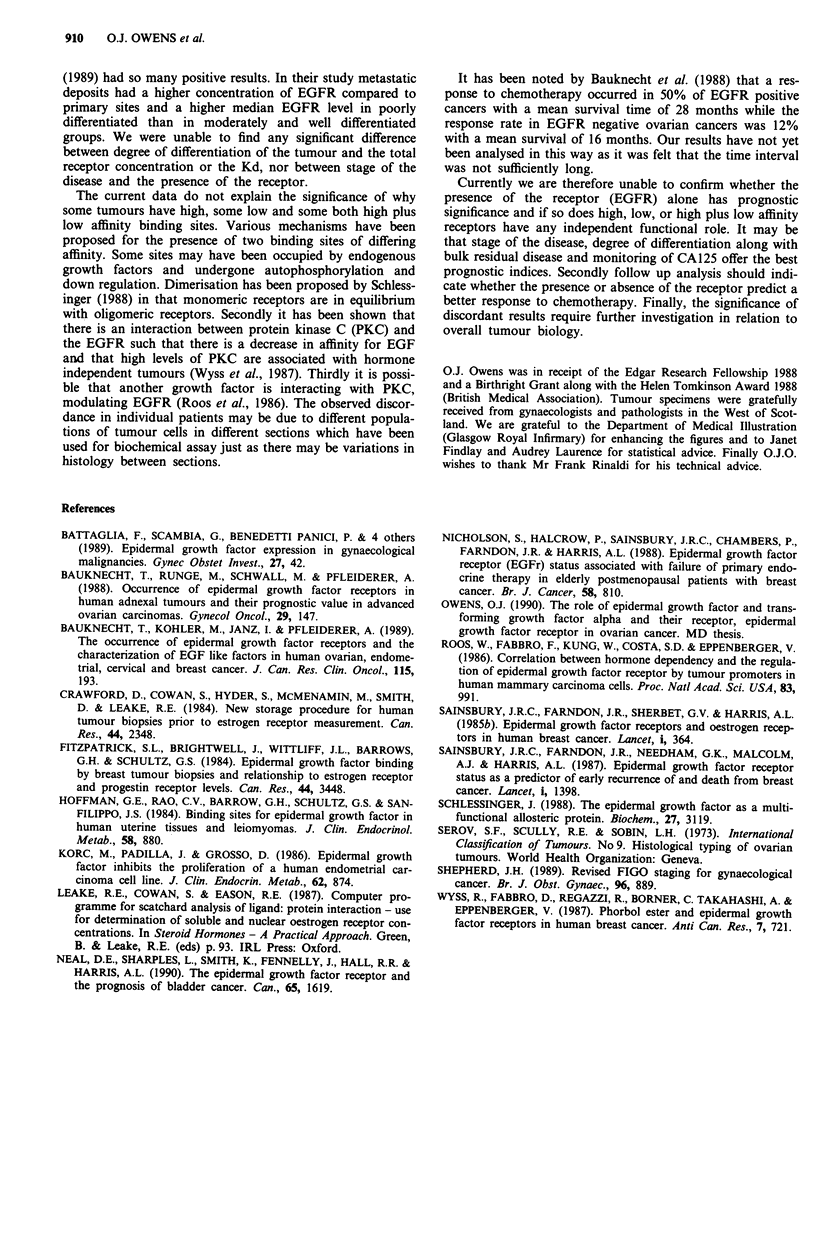# Epidermal growth factor receptors (EGFR) in human ovarian cancer.

**DOI:** 10.1038/bjc.1991.424

**Published:** 1991-11

**Authors:** O. J. Owens, C. Stewart, I. Brown, R. E. Leake

**Affiliations:** Department of Biochemistry, Glasgow University, UK.

## Abstract

Epidermal growth factor receptor can be used as a biological marker in tumours. We examined 199 samples from 150 patients with ovarian cancer first by using a single point screen, then by full Scatchard analysis, over a concentration range between 0.086-16.6 nM. Taking as positive those samples which showed a 20% difference between total binding and non specific binding, the EGFR was present in 39.7% of samples ranging from 36.4% in those tumours which were classified as being mucinous to 47.7% in the undifferentiated group. Thirty-six samples had a low affinity component (Kd greater than 1 nM), 27 had a high affinity component (Kd less than 1 nM) and 16 had both high and low affinity components to the EGFR. There was no statistical difference between degree of differentiation of the tumour and the presence of the EGFR nor between stage of the disease and EGFR presence.


Br. J. Cancer (1991), 64, 907-910 © Macmillan Press Ltd., 1991

### Epidermal growth factor receptors (EGFR) in human ovarian cancer

O.J. Owens[1], C. Stewart[2], I. Brown[3] & R.E. Leake[1]

[1]*Department of Biochemistry, Glasgow University, Glasgow G12 8QQ;* [2]*Department of Pathology, Glasgow Royal Infirmary, Castle Street, Glasgow G4 0SF;* [3]*Department of Pathology, Western General Infirmary, Glasgow G11, UK.*

**Summary** Epidermal growth factor receptor can be used as a biological marker in tumours. We examined 199 samples from 150 patients with ovarian cancer first by using a single point screen, then by full Scatchard analysis, over a concentration range between 0.086–16.6 nM. Taking as positive those samples which showed a 20% difference between total binding and non specific binding, the EGFR was present in 39.7% of samples ranging from 36.4% in those tumours which were classified as being mucinous to 47.7% in the undifferentiated group. Thirty-six samples had a low affinity component (Kd > 1 nM), 27 had a high affinity component (Kd < 1 nM) and 16 had both high and low affinity components to the EGFR. There was no statistical difference between degree of differentiation of the tumour and the presence of the EGFR nor between stage of the disease and EGFR presence.

Epidermal growth factor (EGF) interaction with EGFR results in growth of some epithelial cancer cells *in vitro* and EGFR has been found in both normal and malignant tissue samples. Hoffmann *et al.* (1984) found EGFR in normal human endometrium, while Korc *et al.* (1986) found the receptor in human endometrial carcinoma. Battaglia *et al.* (1989) found that 18 of 24 (75%) ovarian tumours expressed EGFR. Bauknecht *et al.* (1989) reported that 64 of 151 (45%) ovarian cancers displayed the receptor. Others such as Neal *et al.* (1990) found EGFR in 48% of bladder tumours. A significant amount of work has been done with EGFR expression in breast cancer initially by Fitzpatrick *et al.* (1984), more recently by Sainsbury *et al.* (1985 and 1987) and Nicholson *et al.* (1988). In fact Sainsbury *et al.* (1987) found that both EGFR and oestrogen receptor (ER) status could predict prognosis. Patients who were EGFR+/ER− had the worst prognosis and those who were EGFR−/ER+ the best.

To further investigate the role of EGFR in ovarian neoplasia we have examined 150 consecutive patients with ovarian cancer who had at least one sample analysed by a biochemical assay.

### Materials and methods

### *Patient selection, collection and storage of tumour specimens*

Patients were recruited prospectively and included any patient with ovarian cancer. Tumour was either collected fresh, snap frozen in liquid nitrogen and later stored at −70°C or transported on ice in sucrose/glycerol buffer (Crawford *et al.*, 1984) and stored at −20°C until assayed. Occasionally, not only one ovary but both ovaries and omentum were analysed from the same patient. Experiments on placental tissue over time and storage conditions (liquid nitrogen and −70°C or sucrose/glycerol buffer and −20°C) showed that similar results were obtained (Owens, MD thesis, 1990).

### *Preparation of membrane pellet*

Tumour specimens removed from −70°C were allowed to thaw on ice, while those from sucrose/glycerol were re-hydrated in homogenising buffer (see below). The tumour specimen was dried with tissue paper to remove any excess water or buffer. Specimens were washed in ice cold saline. The tumour was bisected and two separate samples of tumour were cut (2–3 mm minimum) from either half, one piece placed in formal saline for pathological analysis and the other in sucrose/glycerol buffer for later immunohistochemical analysis (to be reported later). The remainder of the tumour was used for the biochemical assay. Fresh homogenising buffer was prepared (20 mM Hepes, 2 mM EDTA, 0.5 mM PMSF to pH 7.4). Tumour sections were then cut into small 1 mm blocks and weighed (usually 1 gram), then placed in a centrifuge tube to which was added 5 ml $g^{-1}$ (wet weight) of ice cold homogenising buffer. Tumour was homogenised on ice with an ultra turrax (Janke and Kunkel) with 2 × 15 s bursts at maximum speed but allowing the homogenate to cool between bursts. The homogenate was centrifuged at 1,000 *g* for 10 min. The resulting supernatant was centrifuged at 12,000 *g* for 1 h. The nuclear pellet was resuspended in homogenising buffer and stored at −20°C until required for DNA analysis (Modified Burton). The pellet from the high speed spin was resuspended in 1–2 ml of radioimmunoassay buffer (RIA buffer: 0.2 M $Na_2HPO_4$, 0.2 M $NaH_2PO_4$, 0.1% sodium azide, 0.15 M sodium chloride, 0.1 M EDTA and 0.5% bovine serum albumin to pH 7.4) depending on the initial wet weight (1 ml 500 $mg^{-1}$) and stored at −20°C in 1.5 ml polystyrene tubes (Eppendorfs). Prior to storage each suspension was submitted to glass teflon homogenisation to ensure an even suspension.

### *Single point screen*

Prior to competition assays for determining the presence of the EGFR, eppendorfs were pre-coated with RIA buffer or phosphate buffered saline (PBS in 1% albumin) to reduce EGF sticking to the plastic. All tumour extracts were subjected to a single point screen as follows: Eppendorfs were removed from −20°C and allowed to thaw on ice. The membrane pellet was subjected to glass teflon homogenisation to re-establish a uniform suspension. Labelled mEGF ($^{125}$I NEN) with a specific activity of 90–180 $\mu$Ci $\mu g^{-1}$ was made to a final concentration of 1 nM. The total c.p.m.'s were about 100,000 c.p.m. 100 $\mu l^{-1}$. The non specific binding was determined by adding unlabelled mEGF to give 100 nM final concentration.

To pre-coated eppendorfs was added 50 $\mu$l of cytosol membrane preparation in duplicate, plus 50 $\mu$l of 1 nM $^{125}$I EGF with and without 100 nM unlabelled mEGF to permit calculation of specific binding. All samples were vortexed (Rotamixer) to ensure thorough mixing. Placental membrane was run as a positive control. Incubation was for 2 h at room temperature. The reaction was terminated by adding ice cold RIA buffer (750 $\mu$l) and the eppendorfs were centrifuged at

Correspondence: O.J. Owens, Wards 31/33, Department of Gynaecology, Glasgow Royal Infirmary, Castle Street, Glasgow G4 0SF, UK.
Received 19 March 1991; and in revised form 12 June 1991.

40,000 g in a refrigerated unit at 4°C (Sarstedt) for 10 min. The supernatant was removed with a pasteur attached to a water pump leaving a pellet which was counted on a gamma counter (60% efficiency). The mean of each pair of results was taken and a difference of 20% between total counts and non specific were indicative of a positive single point screen. When positive, a full Scatchard analysis was performed.

*Scatchard analysis*

Multipoint analysis was carried out at 12 points of increasing concentration of labelled EGF (0.086–16.66 nM final concentration). Non-specific binding was ascertained by incubating three aliquots (50 µl) of membrane preparation with the top three concentrations of labelled EGF containing a 100-fold excess of unlabelled EGF. Incubation and termination of the reaction were as for the single point screen.

Data were analysed using an 'in house' programme (adapted from Leake *et al.*, 1987). The programme corrected for non-specific binding using the three competition values (tubes 13–15). It computed the Bound and Bound/Free values. The points were plotted with the aid of a cricket graph programme (1.2) on an Apple Mackintosh computer. The y axis giving the Bound/Free values and the x axis the Bound (Total Receptor Concentration). Each line (or lines) was computed using a minimum of five data points.

*Object of study*

There were three main objectives in this study. Firstly to document the incidence of EGFR in a group of 150 consecutive patients with ovarian cancer both by single point screen and full Scatchard analysis. Secondly to determine if there was any statistical difference between degree of differentiation of the tumour and the presence of the EGFR and finally to see if there was a difference between stage of disease and presence of the EGFR.

*Statistical analysis*

Chi square testing was used for statistical analysis.

### Results

*Placental tissue experiments*

Preliminary experiments, using a human placental membrane fraction, were performed to find out the effect of time on the percentage mEGF bound to placental membrane at 4°C, room temperature and 37°C. Figure 1 shows that maximum binding was achieved at room temperature at 120 min. This formed the basis of the subsequent single point screens and full Scatchard analysis. Figure 2 shows that the addition of 100 fold unlabelled mEGF to EGFR pre-filled with $^{125}$I EGF resulted in the expected reduction in binding. This reached a steady state (at about 20% original binding) after 2 h at room temperature.

*Presence of the receptor*

The high affinity component was taken arbitrarily when the Kd was less than 1 nM and the low affinity component when the Kd was equal to or greater than 1 nM.

*Type of tumour and stage*

The results were grouped and analysed depending on the histological type of tumour (Serov *et al.*, 1973) into five main categories which comprised serous, endometrioid, mucinous, clear cell and undifferentiated sub-types of common epithelial tumours. A further 18 patients were analysed separately. All patients were staged in accordance with the revised FIGO staging for ovarian cancer (Shepherd, 1989). Stage 1 and 2 were subdivided into a, b and c. However, it was not possible to subdivide stage 3 into its various sub stages.

*Results of tumour specimens*

The results are divided into three tables. Table I illustrates the patient characteristics for the major types of common epithelial tumour. The majority of samples fall into the serous group. Sample number is greater than patient number because sometimes tumour was recovered from both ovaries and omentum. The majority of patients were stage 3 and 4. Certainly in the serous group 77.5% of patients presented with stage 3 or 4 disease. Because it is too early to analyse survival (follow up ranges from 1 to 19 months) it was felt unnecessary to subdivide stage 1 and 2 at present.

Table II compares the presence of EGFR in the various groups. EGFR presence varied between 36.4% in the mucinous group and 47.4% in the undifferentiated group. The low affinity receptor was present in 31 of 73 positive samples (42.5%), while the high affinity receptor was present in 26 of 73 (35.6%) and 16 of 73 samples (21.9%) had both high and low affinity receptors.

Table IIIa gives the various ranges and median values for the serous, endometrioid and mucinous group with regard to low, high and low plus high affinity receptor groups. Table IIIb gives the results for the clear cell and undifferentiated group.

**Figure 1** The effect of time and temperature on the percentage of radiolabelled mEGF bound to placental membrane. O, 37°C; ●, room temp; X, 4°C.

**Figure 2** Time course/temperature of displacement of labelled mEGF by excess unlabelled EGF. O, 4°C; ●, room temp; X, 37°C.

**Table I** Patient characteristics

| | *SER* | *ENDO* | *MUC* | *CLEAR* | *UNDIFF* |
|---|---|---|---|---|---|
| Patients | 80 | 19 | 11 | 9 | 13 |
| Samples | 114 | 20 | 11 | 14 | 19 |
| Age range | 25–88 | 43–82 | 45–83 | 52–71 | 43–72 |
| Mean age | 60 | 61.2 | 62.2 | 58.9 | 62.3 |
| *Number of patients per stage of disease* | | | | | |
| Stage 1 | 10 | 9 | 6 | 1 | 1 |
| Stage 2 | 8 | 2 | | 3 | 1 |
| Stage 3 | 41 | 6 | 2 | 2 | 6 |
| Stage 4 | 21 | 2 | 3 | 3 | 5 |

SER: serous, ENDO: endometrioid, MUC: mucinous, CLEAR: clear cell, UNDIFF: undifferentiated.

**Table II** EGFR characteristics

| | *SER* | *ENDO* | *MUC* | *CLEAR* | *UNDIFF* |
|---|---|---|---|---|---|
| Samples | 114 | 20 | 11 | 14 | 19 |
| EGFR + | 46 | 8 | 4 | 6 | 9 |
| % Pos | 40.4 | 40 | 36.4 | 42.8 | 47.4 |
| Low aff. | 17 | 4 | 2 | 2 | 6 |
| High aff. | 18 | 3 | 2 | 1 | 2 |
| High + low | 11 | 1 | | 3 | 1 |

SER: serous, ENDO: endometrioid, MUC: mucinous, CLEAR: clear cell, UNDIFF: undifferentiated. EGFR + : EGFR positive, % Pos: percentage positive, Low aff: low affinity, High aff: high affinity, High + low: high and low affinity receptors.

**Table IIIa** EGFR characteristics

| | *Serous* | *Endometrioid* | *Mucinous* |
|---|---|---|---|
| Low aff Kd (nM) | | | |
| (median) | 2.125 | 1.949 | |
| (range) | 1.02–14.02 | 1.743–15.194 | 5.33 + 8.343 |
| TRC (fmoles $\mathrm{mg^{-1}}$ DNA) | | | |
| (median) | 1398 | 2415 | |
| (range) | 358–9645 | 1527–4893 | 977 + 1300 |
| High aff Kd (nM) | | | |
| (median) | 0.350 | 0.429 | |
| (range) | 0.071–0.942 | 0.025–0.909 | 0.227 + 0.345 |
| TRC (fmoles $\mathrm{mg^{-1}}$ DNA) | | | |
| (median) | 779 | 611 | |
| (range) | 285–1784 | 229–2000 | 984 + 1339 |
| *High plus Low affinity receptors* | | | |
| Low aff Kd (nM) | | | |
| (median) | 6.41 | | |
| (range) | 2.13–17.64 | 7.45 | – |
| TRC (fmoles $\mathrm{mg^{-1}}$ DNA) | | | |
| (median) | 3548 | | |
| (range) | 1092–24667 | 3816 | – |
| High aff Kd | | | |
| (median) | 0.461 | | |
| (range) | 0.079–1.045 | 0.351 | – |
| TRC (fmoles $\mathrm{mg^{-1}}$ DNA) | | | |
| (median) | 1282 | | |
| (range) | 394–10563 | 843 | – |

Low aff Kd: low affinity Kd, high aff Kd: High affinity Kd, TRC: Total receptor concentration.

**Table IIIb** EGFR characteristics

| | *Clear cell* | *Undifferentiated* |
|---|---|---|
| Low aff Kd (nM) | | |
| (median) | | 1.804 |
| (range) | 1.525 + 18.832 | 1.095–7.86 |
| TRC (fmoles $\mathrm{mg^{-1}}$ DNA) | | |
| (median) | | 787 |
| (range) | 1420 + 3801 | 370–3465 |
| High aff Kd (nM) | | |
| (values) | 0.175 | 0.533 + 0.756 |
| TRC (values) | | |
| fmoles $\mathrm{mg^{-1}}$ DNA | 470 | 690 + 740 |
| *High plus Low affinity receptors* | | |
| Low aff Kd (nM) | | |
| (median) | 6.82 | |
| (range) | 2.294–7.025 | 4.919 |
| TRC (fmoles $\mathrm{mg^{-1}}$ DNA) | | |
| (median) | 2972 | |
| (range) | 420-9910 | 6315 |
| High aff Kd | | |
| (median) | 0.145 | |
| (range) | 0.056–0.531 | 0.75 |
| TRC (fmoles $\mathrm{mg^{-1}}$ DNA) | | |
| (median) | 877 | |
| (range) | 147–1928 | 2263 |

Low aff Kd: low affinity Kd, high aff Kd: High affinity Kd, TRC: Total receptor concentration.

Finally the results for those patients (18) which do not fall into the five main categories are reported as follows: The malignant mixed mesodermal group contained seven patients (eight samples) with a mean age of 65.1 years (range 51–79 years). There was one patient with stage 1c, one with stage 2a, one stage 2b and four were stage 3. The EGFR was present in three of eight samples (37.5%) and the receptors were all low affinity with Kd's of 1.95 nM, 1.25 nM and 4.26 nM and with total receptor concentrations of 392, 1,107 and 20,168 fmoles $\mathrm{mg^{-1}}$ DNA. The three cases (three samples) of mixed malignant epithelial tumour were all EGFR negative. One was an endometrioid/mucinous stage 1c and aged 60, the next was a clear cell/serous stage 4 and aged 56 and the last was a serous/mucinous aged 32 and stage 1c. There was one patient with a lipid cell tumour age 64 and stage 3 who had a low affinity receptor (1.91 nM and TRC of 550 foles $\mathrm{mg^{-1}}$ DNA). There were two endometrial stromal sarcomas (three samples), all were EGFR negative (both stage 3). There was one granulosa cell tumour (stage 3) in a 45 year old patient which contained the EGFR (low affinity 4.57 nM with a TRC of 9,796 fmoles $\mathrm{mg^{-1}}$ DNA). There were three germ cell tumours, one a dysgerminoma (stage 1a) which was EGFR negative, a malignant teratoma (stage 2b) in a 76 year old patient which contained the high affinity EGFR (0.412 nM and a TRC of 1,197 fmoles $\mathrm{mg^{-1}}$ DNA) and a yolk sac tumour (two samples) which was EGFR negative (both samples). The latter was a stage 2c and the patient was 27 years. Finally there was one patient with unknown histology age 60, stage 3 and EGFR negative.

Statistical analysis using Chi square testing failed to show any difference between the presence of EGFR and differentiation or stage of disease.

### Discussion

Overall EGFR was present in 39.7% of samples of ovarian tumours. It was felt that the concentration range chosen for the full Scatchard plots (0.086–16.6 nM) would encompass all high and low affinity binding sites. It is difficult to compare results with other authors such as Bauknecht *et al.* (1989) who found EGFR in 45% of ovarian tumours. They used only a single point screen for determining the receptor and if this was positive, they assumed that receptor was therefore present. However we were unable to confirm this point as not all positive single point screens actually have receptors present on full Scatchard analysis. Indeed, less than 70% of positive single point screens contained receptor on full Scatchard analysis using at least five separate data points and a regression coefficient $\geqslant 0.8$. We examined a number of negative single point screen samples and were unable to demonstrate the receptor in any case. Discordant results occurred in up to 25% of samples when more than one specimen from the same patient was analysed. This is probably related to the heterogeneity of ovarian tumours. Battaglia *et al.* (1989) interestingly found that 18 of 24 (75%) primary ovarian samples expressed detectable levels of EGFR. They only used a range of Scatchard points between 0.4–2.6 nM and this range of concentration would not necessarily detect all possible high affinity components nor all low affinity components. It is thus puzzling that Battaglia *et al.*

(1989) had so many positive results. In their study metastatic deposits had a higher concentration of EGFR compared to primary sites and a higher median EGFR level in poorly differentiated than in moderately and well differentiated groups. We were unable to find any significant difference between degree of differentiation of the tumour and the total receptor concentration or the Kd, nor between stage of the disease and the presence of the receptor.

The current data do not explain the significance of why some tumours have high, some low and some both high plus low affinity binding sites. Various mechanisms have been proposed for the presence of two binding sites of differing affinity. Some sites may have been occupied by endogenous growth factors and undergone autophosphorylation and down regulation. Dimerisation has been proposed by Schlessinger (1988) in that monomeric receptors are in equilibrium with oligomeric receptors. Secondly it has been shown that there is an interaction between protein kinase C (PKC) and the EGFR such that there is a decrease in affinity for EGF and that high levels of PKC are associated with hormone independent tumours (Wyss *et al.*, 1987). Thirdly it is possible that another growth factor is interacting with PKC, modulating EGFR (Roos *et al.*, 1986). The observed discordance in individual patients may be due to different populations of tumour cells in different sections which have been used for biochemical assay just as there may be variations in histology between sections.

It has been noted by Bauknecht *et al.* (1988) that a response to chemotherapy occurred in 50% of EGFR positive cancers with a mean survival time of 28 months while the response rate in EGFR negative ovarian cancers was 12% with a mean survival of 16 months. Our results have not yet been analysed in this way as it was felt that the time interval was not sufficiently long.

Currently we are therefore unable to confirm whether the presence of the receptor (EGFR) alone has prognostic significance and if so does high, low, or high plus low affinity receptors have any independent functional role. It may be that stage of the disease, degree of differentiation along with bulk residual disease and monitoring of CA125 offer the best prognostic indices. Secondly follow up analysis should indicate whether the presence or absence of the receptor predict a better response to chemotherapy. Finally, the significance of discordant results require further investigation in relation to overall tumour biology.

O.J. Owens was in receipt of the Edgar Research Fellowship 1988 and a Birthright Grant along with the Helen Tomkinson Award 1988 (British Medical Association). Tumour specimens were gratefully received from gynaecologists and pathologists in the West of Scotland. We are grateful to the Department of Medical Illustration (Glasgow Royal Infirmary) for enhancing the figures and to Janet Findlay and Audrey Laurence for statistical advice. Finally O.J.O. wishes to thank Mr Frank Rinaldi for his technical advice.

### References

BATTAGLIA, F., SCAMBIA, G., BENEDETTI PANICI, P. & 4 others (1989). Epidermal growth factor expression in gynaecological malignancies. *Gynec Obstet Invest.*, **27**, 42.

BAUKNECHT, T., RUNGE, M., SCHWALL, M. & PFLEIDERER, A. (1988). Occurrence of epidermal growth factor receptors in human adnexal tumours and their prognostic value in advanced ovarian carcinomas. *Gynecol Oncol.*, **29**, 147.

BAUKNECHT, T., KOHLER, M., JANZ, I. & PFLEIDERER, A. (1989). The occurrence of epidermal growth factor receptors and the characterization of EGF like factors in human ovarian, endometrial, cervical and breast cancer. *J. Can. Res. Clin. Oncol.*, **115**, 193.

CRAWFORD, D., COWAN, S., HYDER, S., MCMENAMIN, M., SMITH, D. & LEAKE, R.E. (1984). New storage procedure for human tumour biopsies prior to estrogen receptor measurement. *Can. Res.*, **44**, 2348.

FITZPATRICK, S.L., BRIGHTWELL, J., WITTLIFF, J.L., BARROWS, G.H. & SCHULTZ, G.S. (1984). Epidermal growth factor binding by breast tumour biopsies and relationship to estrogen receptor and progestin receptor levels. *Can. Res.*, **44**, 3448.

HOFFMAN, G.E., RAO, C.V., BARROW, G.H., SCHULTZ, G.S. & SANFILIPPO, J.S. (1984). Binding sites for epidermal growth factor in human uterine tissues and leiomyomas. *J. Clin. Endocrinol. Metab.*, **58**, 880.

KORC, M., PADILLA, J. & GROSSO, D. (1986). Epidermal growth factor inhibits the proliferation of a human endometrial carcinoma cell line. *J. Clin. Endocrin. Metab.*, **62**, 874.

LEAKE, R.E., COWAN, S. & EASON, R.E. (1987). Computer programme for scatchard analysis of ligand: protein interaction – use for determination of soluble and nuclear oestrogen receptor concentrations. In *Steroid Hormones – A Practical Approach*. Green, B. & Leake, R.E. (eds) p. 93. IRL Press: Oxford.

NEAL, D.E., SHARPLES, L., SMITH, K., FENNELLY, J., HALL, R.R. & HARRIS, A.L. (1990). The epidermal growth factor receptor and the prognosis of bladder cancer. *Can.*, **65**, 1619.

NICHOLSON, S., HALCROW, P., SAINSBURY, J.R.C., CHAMBERS, P., FARNDON, J.R. & HARRIS, A.L. (1988). Epidermal growth factor receptor (EGFr) status associated with failure of primary endocrine therapy in elderly postmenopausal patients with breast cancer. *Br. J. Cancer*, **58**, 810.

OWENS, O.J. (1990). The role of epidermal growth factor and transforming growth factor alpha and their receptor, epidermal growth factor receptor in ovarian cancer. MD thesis.

ROOS, W., FABBRO, F., KUNG, W., COSTA, S.D. & EPPENBERGER, V. (1986). Correlation between hormone dependency and the regulation of epidermal growth factor receptor by tumour promoters in human mammary carcinoma cells. *Proc. Natl Acad. Sci. USA*, **83**, 991.

SAINSBURY, J.R.C., FARNDON, J.R., SHERBET, G.V. & HARRIS, A.L. (1985*b*). Epidermal growth factor receptors and oestrogen receptors in human breast cancer. *Lancet*, **i**, 364.

SAINSBURY, J.R.C., FARNDON, J.R., NEEDHAM, G.K., MALCOLM, A.J. & HARRIS, A.L. (1987). Epidermal growth factor receptor status as a predictor of early recurrence of and death from breast cancer. *Lancet*, **i**, 1398.

SCHLESSINGER, J. (1988). The epidermal growth factor as a multifunctional allosteric protein. *Biochem.*, **27**, 3119.

SEROV, S.F., SCULLY, R.E. & SOBIN, L.H. (1973). *International Classification of Tumours*. No 9. Histological typing of ovarian tumours. World Health Organization: Geneva.

SHEPHERD, J.H. (1989). Revised FIGO staging for gynaecological cancer. *Br. J. Obst. Gynaec.*, **96**, 889.

WYSS, R., FABBRO, D., REGAZZI, R., BORNER, C. TAKAHASHI, A. & EPPENBERGER, V. (1987). Phorbol ester and epidermal growth factor receptors in human breast cancer. *Anti Can. Res.*, **7**, 721.